# Evaluation of a public awareness campaign for dementia risk reduction in the Netherlands: a mixed methods study

**DOI:** 10.1186/s12889-025-25676-8

**Published:** 2025-12-12

**Authors:** Dominique Paauw, Irene Heger, Dorothee Horstkötter, Niels Janssen, KlaasJan Hajema, Sandra Kuiper, Anne Loyen, Judith Bouwmeester, Anja Lens, Judith Helmink, Françoise Schütz, Kay Deckers, Sebastian Köhler

**Affiliations:** 1 Department of Psychiatry and Neuropsychology, Alzheimer Centre Limburg, Maastricht University, Maastricht, The Netherlands; 2 Mental Health and Neuroscience Research Institute (MHeNs), Maastricht University, Maastricht, The Netherlands; 3 Department of Health Ethics and Society, Maastricht University, Maastricht, The Netherlands; 4 GGD Zuid-Limburg, Heerlen, The Netherlands; 5 GGD West-Brabant, Breda, The Netherlands; 6 GGD Flevoland, Lelystad, The Netherlands; 7 GGD Zuid-Holland Zuid, Dordrecht, The Netherlands; 8 Plicare, Zoetermeer, The Netherlands; 9 GGD Hart Voor Brabant, Tilburg, The Netherlands; 10 GGD Regio Utrecht, Zeist, The Netherlands

**Keywords:** Awareness, Brain health, Dementia, Lifestyle, Prevention, Campaign

## Abstract

**Background:**

About 45% of dementia cases are attributable to known modifiable risk factors, yet public awareness remains low. The public awareness campaign “We are the medicine ourselves” (2018–2023) aimed to increase public awareness of dementia risk reduction in nine regions. This mixed methods study evaluated the campaign’s effectiveness in reaching individuals aged 40–75 years and explored facilitators and barriers to successful implementation.

**Methods:**

Cross-sectional online surveys were conducted in independent samples of community-dwelling individuals aged 40–75 years before (*n* = 4,981) and after (*n* = 3,379) the campaign to assess awareness of dementia risk reduction, knowledge of dementia risk and protective factors, and campaign exposure. Differences between pre- and post-campaign samples were assessed using χ^2^-tests for categorical variables and independent t-tests for continuous variables. Adjusted effects were estimated using probit regression for binary outcomes and linear regression for continuous outcomes, controlling for region, age, gender, educational level, and self-reported knowledge of dementia. Semi-structured interviews with 21 campaign coordinators and stakeholders explored facilitators and barriers for implementation.

**Results:**

No significant difference in awareness was found between the pre-campaign (54.6% aware) and post-campaign (55.7% aware) samples (adjusted probit regression: z = 0.97, *p* = 0.334). Knowledge of risk and protective factors modestly increased (from 5.3 to 5.5; B = 0.18, 95% confidence interval: 0.04 – 0.33, *p* = 0.013). Self-reported campaign exposure was associated with higher awareness, better knowledge of risk and protective factors, and greater motivation to adopt brain-healthy lifestyle changes. Implementation barriers included limited financial resources, difficulty reaching younger individuals (40–60 years), and limited engagement with regional stakeholders. Facilitators included the campaign framework and strong local networks.

**Conclusions:**

While the multi-regional campaign did not lead to a general increase in population-level awareness of dementia risk reduction, it modestly improved knowledge of specific dementia risk and protective factors.

**Supplementary Information:**

The online version contains supplementary material available at 10.1186/s12889-025-25676-8.

## Background

Dementia is a clinical syndrome characterised by cognitive decline that interferes with daily functioning, leading to dependency, disability, and death [1]. In 2019, approximately 57 million individuals were living with dementia worldwide, and this number is projected to almost triple to 153 million by 2050 because of population ageing and increased life expectancy [2], causing a major public health challenge [3].

Hence, a current focus is on reducing dementia risk through lifestyle modifications and treatment of conditions that increase risk. A substantial number of dementia cases are potentially attributable to 14 modifiable risk factors: less education in early life; hearing loss, high LDL cholesterol, depression, traumatic brain injury, physical inactivity, diabetes, smoking, hypertension, obesity, and excessive alcohol consumption in midlife; and social isolation, air pollution, and visual loss in later life [4]. Most of these factors have also been incorporated into dementia risk reduction guidelines developed by the World Health Organization [5].

Yet, the general population is largely unaware of the potential of dementia risk reduction [6], even in European high-income countries such as the Netherlands (44%) and Flanders-Belgium (35%) [7–9], with higher levels found in recent studies in Norway (70%), Germany (68%), and Denmark (67%) [10–12]. Consistently, knowledge of modifiable risk factors was limited, and survey participants (> 70%) expressed a need for more information.

To inform the public, public awareness campaigns have been conducted in Europe, with conflicting results [7, 9, 12, 13]. In Ireland and Flanders, awareness increased following a mass media campaign [7, 13], but this was not observed in Denmark and the Netherlands [9, 12]. The Dutch campaign “We zijn zelf het medicijn” (“We are the medicine ourselves”) was initially conducted in the Province of Limburg from March 2018 to February 2019 and comprised a mass media approach, public outreach activities, and a program for regional supporters (“campaign friends”). The campaign did not lead to population-level change in awareness, but dementia risk and protective factors were more often correctly identified after the campaign, and awareness was higher in those exposed to the campaign [9, 12]. Since then, the campaign has been adopted and evaluated in other regions across the Netherlands.

The aim of this mixed methods study was to assess whether this multi-regional campaign was effective in reaching middle-aged to older community-dwelling individuals (40–75 years) by pooling data from 9 campaigns conducted so far, and to assess the processes that might have hindered or facilitated successful implementation in order to inform future campaign roll-outs through interviews with regional campaign stakeholders.

## Methods

### Public awareness campaign

“We zijn zelf het medicijn” was developed by the Alzheimer Centre Limburg of Maastricht University and the Maastricht University Medical Centre +, in consultation with the two regional Municipal Health Service (MHS) offices and the Department of Health Promotion of Maastricht University [8, 9]. It aimed to increase awareness of modifiable risk factors for dementia in individuals aged 40–75 years.

The campaign focused on three main messages: ‘eat healthy’, ‘exercise regularly’, and ‘stay curious’. It was delivered by regional MHS staff in collaboration with local stakeholders (e.g., municipality staff, general practitioners). Multiple communication channels were used, including weekly public events (e.g., lectures, information markets, workshops), social media platforms (e.g., Facebook, LinkedIn, Twitter [now X]), freely downloadable campaign materials (e.g., posters, leaflets; see Supplementary File 1), a campaign website with information, news, local events, and the MyBraincoach app. This app was developed and offered free of charge to advise people on a brain-healthy lifestyle [14]. The content was based on the 12 modifiable risk and protective factors for dementia included in the LIBRA (LIfestyle for BRAin health) score: high cognitive activity, physical inactivity, healthy diet, (midlife) hypertension, depression, smoking, diabetes, low-to-moderate alcohol intake, (midlife) obesity, hypercholesterolemia, coronary heart disease, and chronic kidney disease [15–18].

Fidelity to the campaign was maintained through standardised campaign messages and central coordination by the research team, while allowing regions to adapt the campaign to local context and preferences. Adaptations included variations in campaign duration, activities, intensity, communication channels, and culturally relevant (evidence-based) examples/translations to enhance engagement. This study evaluates nine regional campaigns that have been executed so far, i.e., Limburg (2 subregions), West-Brabant, Haaglanden, Zuid-Holland Zuid, Flevoland, Hart voor Brabant, Utrecht, and the municipality of Zoetermeer. The initial rollout was in the MHS regions Noord-Limburg and Zuid-Limburg in 2018 (collectively referred to as “Limburg” in this paper).

### Campaign evaluation framework

The evaluation was guided by the RE-AIM framework, which focuses on Reach, Effectiveness, Adoption, Implementation, and Maintenance [19]. This framework informed the design of the evaluation, including which outcomes to measure, how to assess participant engagement, and how to monitor adherence to the implementation plan. Regional adaptations and differences in campaign delivery were considered to understand how local context influenced both implementation and outcomes. RE-AIM enabled a systematic evaluation of both the campaign’s impact on awareness and knowledge of dementia risk reduction, as well as the delivery across regions.

### Study population and procedures

#### Cross-sectional survey study

Each regional campaign included separate pre- and post-campaign survey samples among community-dwelling middle-aged to older (40–75 years) inhabitants (see Results section, Table 1). This age range was based on epidemiological studies suggesting that the associations between risk factors and dementia are most pronounced in midlife [20, 21]. Participants were recruited from local MHS research panels and invited via email to complete the online survey. In general, they had a two-week period to complete the survey, with a reminder email sent after two weeks. No incentives were provided. The survey required approximately 10–15 min to complete. Each panel member received a unique survey link, allowing only a single submission per link. Responses were collected anonymously, and data were securely stored on encrypted servers, accessible only to authorized members of the research team. Sampling within each panel was random but stratified by age, sex, and municipality to ensure representativeness. Sample sizes were determined by the available research panel size in each region and aimed to provide sufficient data to assess changes in dementia awareness, while allowing for stratified analyses by age, gender and level of education. Participants were eligible if they were aged between 40 and 75 years. Individuals who participated in the pre-campaign survey were not eligible for the post-campaign survey to avoid campaign-priming and potential learning effects. For quantitative analyses, datasets from the regional campaigns were harmonised and merged. Participants were excluded if they discontinued the survey prior to answering the awareness question. In addition, data from MHS regions Haaglanden and Flevoland, as well as from the municipality of Zoetermeer, were excluded due to limited survey data.

#### Semi-structured interviews

Local campaign coordinators and stakeholders were invited to participate in interviews to evaluate the implementation process. Stakeholders were individuals actively involved in the campaign, ranging from displaying campaign materials to organising campaign events. They were selected to ensure a broad representation of stakeholder roles (e.g., general practitioner, communication advisor, library volunteer). Of the 22 invited individuals, 21 participated and one declined due to a change in employment. Interviews were conducted online via Microsoft Teams by two members of the research team (D.P., I.H.) between July 2023 and February 2024. D.P. was a PhD candidate with a master’s degree, and I.H. was a postdoctoral researcher with a PhD; both are female and had training in qualitative research methods. Neither interviewer had a prior relationship with participants. Participants were invited by email, with a reminder sent after two weeks. Upon acceptance, participants received a digital information letter and informed consent form. At the start of each interview, participants were informed about the interviewers’ roles, credentials, and the purpose of the study. The interviewers had a professional interest in dementia prevention and public awareness campaigns and reflected on their assumptions and potential biases during data collection and analyses to minimise influence on participants’ responses. Interviews were audio-recorded and securely stored on encrypted servers. After transcribing, the audio recordings were deleted. Notes were made during and immediately after each interview to capture initial reflections. Recruitment continued until data saturation was reached, indicating that no new insights were obtained during the final interviews, with at least one participant recruited per region. Each interview lasted approximately 30–60 min.

### Measures

#### Cross-sectional survey study

Data were collected using self-administered online cross-sectional surveys conducted before and after the campaign. The pre- and post-campaign surveys were identical in content, encompassing three sections: demographics, dementia knowledge, and dementia risk awareness. The post-campaign survey included an additional section assessing participants’ exposure to the campaign. The pre-campaign survey contained 23 items, while the post-campaign survey contained 32 items in total (see Supplementary File 2 for the complete pre- and post-campaign surveys).

Demographics included age, gender and educational level. Age was self-reported and categorised into 40–64 and 65–75 years. Educational level was categorised according to the Dutch system into low, middle, and high. Dementia knowledge was measured using a single self-reported item and dichotomised into “Good” knowledge, comprising the answer options “A great deal,” “Quite a lot,” and “Some”, and “Poor” knowledge, comprising “Not very much”, “Nothing at all”, and “I don’t know”. The primary outcome, population-level change in awareness of dementia risk reduction, was assessed as the difference in the probability of participants rejecting the statement “There is nothing anyone can do to reduce their dementia risk” in the pre-campaign and post-campaign samples. Awareness was coded as present if participants (strongly) disagreed with the statement. The answer option “Neither agree nor disagree” was coded as unaware. Secondary outcomes included knowledge of the three campaign messages (cognitive activity, physical activity, and healthy diet) and of the individual dementia risk and protective factors, as included in the well-validated LIBRA score [15–18]. These were coded in the same manner, with each correctly identified factor coded as “aware” (1) and each incorrectly identified or unidentified factor coded as “unaware” (0). A sum score was calculated as the average number of correctly identified factors. Additionally, participants also answered questions concerning their interest in receiving information on the relationship between lifestyle and brain health, preferred sources of information, and perceived barriers to engaging in a brain-healthy lifestyle. In the post-campaign survey, participants answered questions concerning their exposure to the campaign, recognition of campaign material, whether they downloaded the MyBraincoach app, self-reported increased awareness of the relationship between lifestyle and brain health, and implemented lifestyle changes to improve their brain health.

#### Semi-structured interviews

The interview guide was divided into seven topics: scope and goal of the campaign, campaign preparations, positive aspects, areas for improvement, engagement of stakeholders, campaign materials, and reactions from the public (see Supplementary File 3).

### Analyses

#### Quantitative analysis

Descriptive statistics were calculated to characterise the pre- and post-campaign samples. Categorical variables are presented as frequencies and percentages. Continuous variables are presented as means and standard deviations (Table 2). Differences between pre- and post-campaign samples and the association between the effects and exposure to the campaign in different sociodemographic groups were assessed using χ^2^-tests for categorical variables and independent t-tests for continuous variables. In case of differences in sociodemographic variables between groups of interest (e.g., pre- vs. post-campaign samples; exposed vs. non-exposed individuals), probit regression analysis (for binary outcomes) and linear regression (for continuous outcomes) were used, including adjustment for differences between MHS regions, age, gender, educational level, and self-reported knowledge of dementia. Reference groups were Limburg (MHS region), male (gender), low (educational level), and good (self-reported knowledge of dementia).

Stratified analyses were conducted to examine whether the effects of the campaign differed across subgroups, including region, age, gender, and educational level. Interaction analyses were performed by including multiplicative interaction terms between survey period (pre- vs. post-campaign) and these subgroup variables in the regression models, allowing assessment of whether changes in awareness varied by subgroup.

Combined overall reach was defined as the post-campaign recognition of at least one campaign element (e.g., campaign, slogan, MyBraincoach app, materials). A composite variable was generated, which assigns a value of 1 if at least one of the elements was recognised, ensuring that participants were counted only once regardless of the number of elements recognised. Campaign intensity was measured by two members of the research team (D.P., I.H.) based on the number of organised activities and public media posts during the campaign period.

To provide context for the primary outcome, a post-hoc detectable-effect analysis was conducted. Using the available pre- and post-campaign sample sizes, a two-tailed significance level of 0.05, and 80% power, the minimum detectable difference in the primary outcome measure was calculated. All analyses were performed using Stata 17.0 (StataCorp, College Station, Texas, USA), with the level of statistical significance set at 0.05 in two-tailed tests.

#### Qualitative analysis

The interviews were de-identified and transcribed verbatim in Dutch by one author (D.P.). Two authors independently conducted an inductive thematic analysis using ATLAS.ti 23.2.3. Codes were developed iteratively: each coder initially reviewed a subset of transcripts to generate preliminary codes, after which the coding frame was refined through repeated transcript review and research team discussions. In the case of discrepancies, additional meetings were held with two other members of the research team (S.K., K.D.) to reach consensus.

Once coding was complete, codes were grouped into broader categories based on conceptual similarity and relevance to the research questions. From these categories, common themes were derived. All authors were consulted on the final theme definitions and interpretations. For verification and data quality purposes [22], a member check was conducted by sending a summary of the finalized themes and interpretations to the interviewees (see Supplementary File 4). They were encouraged to provide feedback within four weeks of receiving the document (no changes suggested). A detailed coding tree, including all codes, categories, and themes, is provided in Supplementary File 5.

### Ethical approval

All participants were informed about the study and provided electronic (online survey) or written (interview) consent before participation. The pre- and post-campaign surveys of Limburg were approved by the Ethics Review Committee Psychology and Neuroscience of Maastricht University (reference number 177–07-03–2017). The other pre- and post-campaign surveys were administered in MHS research panels or by the municipality of Zoetermeer and were not subject to ethical approval. The Ethics Review Committee Faculty of Health, Medicine and Life Sciences (FHML-REC) of Maastricht University approved the semi-structured interviews (reference number FHML-REC/2023/023).

## Results

### Pre- and post-campaign surveys

An overview of the campaign characteristics is provided in Table 1. Campaign durations varied between 6 and 15 months. Implementation took place before, during, or after the COVID-19 pandemic, and campaign intensity ranged from low (+) to high (+++).

**Table 1:** Characteristics of campaign regions

Campaign region (year of launch)	Campaign duration	Timing COVID-19 pandemic	Campaign intensity (+/+ +/+ + + ^1^)
Limburg (2018)	10 months	Before	**+++**
West-Brabant (2021)	9 months	During	**+++**
Haaglanden (2021)	9 months	During	**++**
Municipality of Zoetermeer (2022)	12 months	During	**+++**
Zuid-Holland Zuid (2022)	12 months	During	**+++**
Flevoland (2022)	12 months	During	**+++**
Hart voor Brabant (2023)	15 months	After	**+**
Utrecht (2023)	6 months	After	**++**

^1^low campaign intensity (+), medium campaign intensity (+ +), high campaign intensity (+ + +), based on the number of organised campaign activities and public media posts during the campaign period

See Supplementary File 6 for an overview of all campaign regions. The MHS regions Haaglanden and Flevoland, as well as the municipality of Zoetermeer, were excluded from the quantitative analyses due to limited survey data.

A total of 8,360 people participated in the surveys, with 4,981 participants in the pre-campaign survey and 3,379 participants in the post-campaign survey. Because data were collected through MHS research panels, information on the total number of individuals invited, unique visitors, and view, participation, or completion proportion was not available. Consequently, response rates could not be calculated. Despite the random sampling, due to variations across regions, the two samples differed significantly in terms of age, educational level, and self-reported knowledge of dementia. The post-campaign sample included a significantly higher proportion of participants aged 40–64 years and participants with a middle or high level of education (Table 2). Some regions included questions as being optional, leading to missing data for gender (*n* = 22), educational level (*n* = 182), and self-reported knowledge of dementia (*n* = 1). Some individuals in the post-campaign survey from West-Brabant had participated in the pre-campaign survey and were excluded.

**Table 2:** Sample demographic characteristics and self-reported knowledge of dementia in 40–75-year-old adults from campaign regions in the Netherlands, surveyed 2017–2023

Sample characteristics	Pre-campaign *n* = 4,981	Post-campaign *n* = 3,379	*p-value*
MHS region, n (%)			< 0.001
Limburg	570 (11.4%)	590 (17.5%)	
West- Brabant	1738 (34.9%)	357 (10.6%)	
Zuid-Holland Zuid	549 (11.0%)	484 (14.3%)	
Hart voor Brabant	370 (7.4%)	317 (9.4%)	
Utrecht	1754 (35.2%)	1631 (48.3%)	
Age (years), mean (SD^1^)	60.3 (9.6)	59.1 (9.3)	< 0.001
Female, n (%)	2668 (53.6%)	1869 (55.6%)	0.164
Educational level, n (%)			0.001
Low	870 (17.9%)	491 (14.8%)	
Middle	1367 (28.2%)	989 (29.7%)	
High	2614 (53.9%)	1847 (55.5%)	
Self-reported knowledge of dementia, n (%)			0.534
Good	4193 (84.2%)	2862 (84.7%)	
Poor	787 (15.8%)	517 (15.3%)	

^1^Standard deviation (SD). MHS = Municipal Health Service. P-values for categorical variables (MHS region, gender, educational level, self-reported knowledge of dementia) were derived from χ^2^-tests. P-values for the continuous variable (age) were derived from independent t-tests

#### Pre-post campaign differences

In unadjusted analyses, awareness of dementia risk reduction was 55.0% (95% confidence interval (CI): 53.6% – 56.4%) in the pre-campaign sample and 54.3% (95% CI: 52.7% – 56.0%) in the post-campaign sample. The unadjusted difference was −0.7 percentage points, indicating no significant change in awareness. After adjusting for differences in MHS region, age, gender, educational level, and self-reported knowledge of dementia, the predicted probability of awareness was 54.6% (95% CI: 53.1% – 56.1%) pre-campaign and 55.7% (95% CI: 54.0% – 57.5%) post-campaign. The adjusted difference was 1.1 percentage points (B = 0.03, probit z = 0.96, 95% CI: −0.03–0.09, *p* = 0.336), also showing no significant change in awareness. A post-hoc detectable-effect analysis indicated that the smallest difference reliably detectable difference in awareness was approximately 3.1 percentage points. The observed unadjusted and adjusted differences were below this threshold, incidicating that the study was underpowered to detect such small changes.

**Fig. 1:**
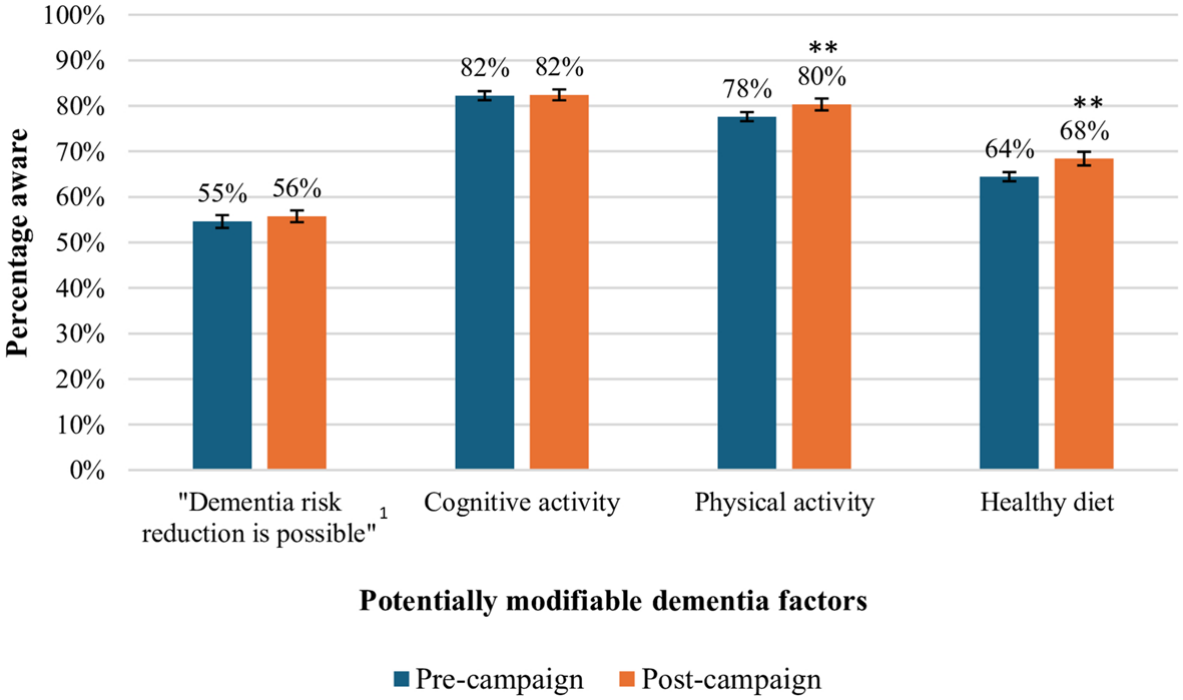
Pre-post comparison awareness dementia risk reduction and campaign messages. Pre-campaign (*n* = 4,981) and post-campaign (*n* = 3,379) comparison of the participants agreeing with the statement that dementia risk reduction is possible, and percentage identifying the three campaign messages. ^1^Original statement presented to participants: “There is nothing anyone can do to reduce their dementia risk”. **: significant difference compared to the pre-campaign sample (*p* < 0.01). All comparisons were adjusted for differences in Municipal Health Service (MHS) region, age, and educational level between the pre- and post-campaign samples. Error bars indicate 95% confidence intervals

Interaction analysis revealed a significant pre-post difference in awareness between the MHS regions Limburg and West-Brabant (B = 0.21, probit z = 1.97, 95% CI: 0.00–0.41,* p* = 0.049), but stratified analyses showed no significant difference in awareness for any MHS region (results not shown). Interaction analyses showed no significant pre-post difference in the level of awareness by age (B = 0.00, probit z = −0.73, 95% CI: −0.01–0.00, *p* = 0.465), educational levels (X^2^ (2) = 2.51, *p* = 0.285), gender (X^2^ (2) = 0.65, *p* = 0.721), and self-reported knowledge levels of dementia (B = 0.05, probit z = 0.62, 95% CI: −0.10–0.20,* p* = 0.537). Figure 1 presents a pre-post campaign comparison of dementia risk reduction awareness and identification of the three campaign messages.

**Fig. 2:**
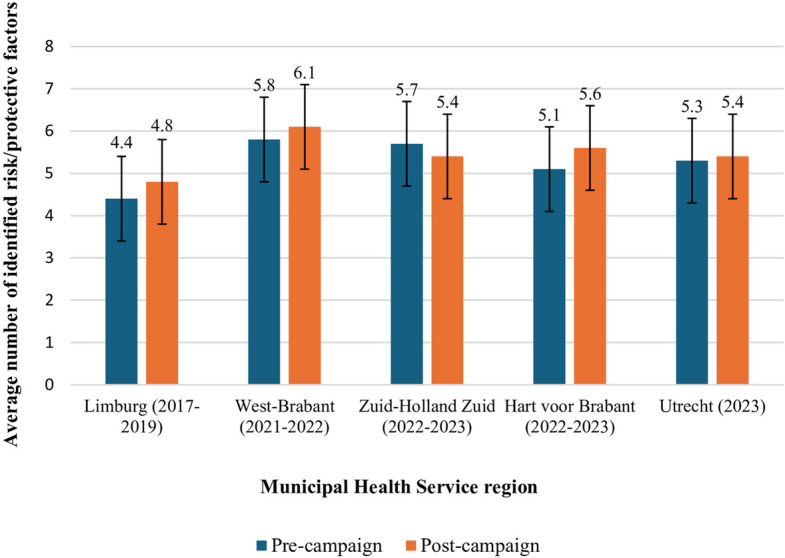
Pre-post comparison correctly identified risk and protective factors. Pre-campaign (*n* = 4,981) and post-campaign (*n* = 3,379) comparison of the average number of correctly identified dementia risk and protective factors per Municipal Health Service (MHS) region. All comparisons were adjusted for differences in MHS regions, age, and educational level between the pre- and post-campaign samples. Error bars indicate 95% confidence intervals

The average number of correctly identified risk and protective factors (Fig. 2) was modestly higher post-campaign compared to pre-campaign (from 5.3 to 5.5; B = 0.18, 95% CI: 0.04–0.33, *p* = 0.013). Interaction analysis showed a significant pre-post difference between the MHS regions Limburg and Zuid-Holland Zuid (B = −0.61, 95% CI: −1.16 – −0.05, *p* = 0.032), but stratified analyses again showed no significant difference in any MHS region (results not shown). Interaction analysis showed a pre-post difference in the average number of correctly identified risk and protective factors between educational levels (F(2, 7930) = 3.04, *p* = 0.048). Further stratified analyses revealed a significant increase in the average number of correctly identified risk and protective factors in the middle-educated group (B = 0.31, t = 2.26, 95% CI: 0.04–0.58, *p* = 0.024), but no significant differences in the lower-educated (B = 0.10, t = 0.55, 95% CI: −0.26–0.46, *p* = 0.586) and higher-educated groups (B = 0.14, t = 1.40, 95% CI: −0.06–0.34, *p* = 0.162). Interaction analysis showed no significant pre-post difference in the average number of correctly identified risk and protective factors by age (B = 0.00, 95% CI: −0.02–0.01,* p* = 0.609), gender (F(2, 8057) = 1.22, *p* = 0.295), and self-reported knowledge levels of dementia (B = −0.15, 95% CI: −0.54–0.25, *p* = 0.470). Results of the pre-campaign sample regarding self-reported knowledge of dementia, awareness, knowledge of individual risk/protective factors, needs, wishes, and barriers are presented in Supplementary File 7.

#### Campaign reach

Among post-campaign participants who answered campaign exposure questions, 12.5% (*n* = 349, 95% CI: 0.11–0.14) had heard about the campaign, 15.9% (*n* = 500, 95% CI: 0.15–0.17) recognised the campaign slogan, 12.8% (n = 334, 95% CI: 0.12–0.14) had heard of the MyBraincoach app, and 17.3% (*n* = 538, 95% CI: 0.16–0.19) recognised at least one campaign material, yielding a combined overall reach of 34.0% (95% CI: 0.32–0.36). Women stated more often to have heard about the MyBraincoach app compared to men (women: 14.9%, 95% CI: 0.13–0.17; men: 10.0%, 95% CI: 0.08–0.12; X^2^(1) = 14.08, *p* = 0.001). Participants aged ≥ 65 years more frequently recognised the campaign slogan (65–75 years: 19.3%, 95% CI: 0.17–0.22; 40–64 years: 13.9%, 95% CI: 0.12–0.16; X^2^(1) = 16.05, *p* ≤ 0.001). Those with low or middle education had heard more often about the campaign (low: 14.1%, 95% CI: 0.11–0.18; middle: 14.5%, 95% CI: 0.12–0.17; high: 11.0%, 95% CI: 0.10–0.13; X^2^(1) = 7.01, *p* = 0.030), the campaign slogan (low: 19.7%, 95% CI: 0.16–0.24; middle: 16.0%, 95% CI: 0.14–0.19; high: 14.7%, 95% CI: 0.13–0.16; X^2^(1) = 7.00, *p* = 0.030), and recognised campaign materials more often than highly educated participants (low: 20.2%, 95% CI: 0.17–0.24; middle: 21.0%, 95% CI: 0.18–0.24; high: 14.6%, 95% CI: 0.13–0.16; X^2^(1) = 20.15, *p* ≤ 0.001).

#### Exposure to the campaign and level of awareness

Exposure was significantly higher among older individuals, women, and lower/middle-educated participants. Further analyses were therefore adjusted for these factors, next to MHS region. In the post-campaign sample, awareness of dementia risk reduction was significantly higher among exposed than non-exposed individuals (60.5% vs. 51.6%; probit z = 4.74, *p* ≤ 0.001). Exposed individuals also identified significantly more dementia risk and protective factors correctly than those unexposed (6.1 vs. 5.0; B = 1.12, 95% CI: 0.88–1.35, *p* ≤ 0.001). Furthermore, exposed participants more often reported lifestyle changes to improve their brain health compared to non-exposed participants (36.5% vs. 23.5%, probit z = 7.54, *p* ≤ 0.001), primarily being more physically active (51.4%), eating healthier (45.1%), and improving weight management (33.7%).

### Semi-structured interviews

Six campaign coordinators and fifteen stakeholders, representing all nine regions, participated in the interviews (Table 3). Our analysis identified five main themes related to facilitators and barriers for campaign implementation. Table 4 presents an overview of all implementation facili­tators and barriers.

**Table 3:** Characteristics of interviewees

Characteristics	Number of participants (*n* = 21)
Gender (*n*)	
Male	2
Female	19
MHS region (*n*)	
Limburg	6
West-Brabant	4
Haaglanden	1
Municipality of Zoetermeer	4
Zuid-Holland Zuid	3
Flevoland	1
Hart voor Brabant	1
Utrecht	1
Role during the campaign (*n*)	
Campaign coordinator	6
Stakeholder	15

#### Campaign framework, preparation, and local network

The pre-existing framework of the campaign, consisting of the campaign website, the MyBraincoach app, and the available campaign materials, was seen as a facilitator for the execution of the campaign.

#### “We set up the campaign quite quickly […] that was possible because the campaign already existed, of course.” (R21, campaign coordinator)

Multiple campaign coordinators mentioned that adequately preparing the municipalities is a facilitator, as it clarifies expectations and enables them to contribute effectively to the campaign.

*“I think that there is still some gain to be made by preparing the municipalities in advance. We really expect communication and commitment to the campaign within the municipality and the town hall. Perhaps we could have focused more on this in the beginning so that they could already figure out how they can arrange that and what they can or cannot do.” (R7, campaign coordinator).*

Another facilitator mentioned by almost all interviewees was the opportunity to collaborate with other (ongoing) initiatives (e.g. regional dementia networks) by making use of their networks and events for spreading the campaign message. Involving welfare institutions was also considered helpful by some. A few stakeholders suggested deploying (language) ambassadors, i.e., individuals with strong networks within the region or specific target groups, to reach the underrepresented groups, such as people aged 40–60 years, those with limited health literacy, migration backgrounds, or language barriers.

*“You could also consider working with a certain ambassador, for example, those who are more active within the age group you want to reach. This also applies to those with low socioeconomic position.” (R19, communication advisor).*

Campaign coordinators recognized that having a strong network of diverse stakeholders is a facilitator, as well as having local campaign friends who actively spread the campaign message through their own channels. Several campaign coordinators had difficulties with finding campaign friends who would proactively engage in the campaign. Some potential campaign friends expected reciprocal benefits in exchange for their commitment, which was often not feasible.

The strong evidence-based foundation of the campaign was acknowledged by both campaign coordinators and stakeholders as a facilitator. In addition, the campaign materials were unanimously well-received as highly appealing.

#### Limited resources, COVID-19, and sustaining the campaign

A limited campaign budget was frequently mentioned as a barrier by the campaign coordinators. Governmental funding requests were, in most cases, not awarded, urging some campaign coordinators to reallocate time from their involvement in other healthy ageing projects.

*”…Our team coordinated with the municipalities in our region, agreeing that they could allocate a few hours per week to the rollout of the campaign. In that regard, hours had also been arranged in advance. Additionally, I was able to contribute additional effort, as my focus on healthy ageing aligned closely with the campaign’s objectives…” (R4, campaign coordinator)*‬‬‬‬‬‬‬‬‬‬‬‬‬‬

Another barrier highlighted by campaign coordinators and stakeholders was the COVID-19 pandemic. The implementation of COVID-19 restrictions curtailed the organisation of on-site campaign events. Additionally, substantial financial resources were diverted towards the pandemic response, resulting in diminished funding for other MHS campaigns. Conversely, the pandemic prompted the professionalization of online events (e.g. webinars), allowing for outreach to larger groups of people.

#### “We wanted to launch the campaign, but then home isolation became mandated, and we weren’t allowed to do any group activities.” (R3, project employee)

Some campaign coordinators expressed a desire to continue the campaign beyond the official campaign period, though they were uncertain about how to achieve this.

*“We conducted the campaign this year, and I think we should continue this for another year, because it is not the case that you have immediately reached everyone and everything after one campaign period. That might also be something to consider… so we are looking into how to continue and what is a useful way to do that.” (R21, campaign coordinator).*

Conversely, most stakeholders indicated a desire to continue using the campaign materials in their region.

#### Engagement of the younger segment of the target group

Both campaign coordinators and stakeholders expressed the difficulty of reaching the younger part of the target group (40–60 years). Nearly all campaign coordinators and stakeholders noticed that predominantly older people, particularly those interested in the topic of dementia, visited public campaign activities.

*“People often think of the elderly when they think of dementia, and they immediately think of the target group 65* + *. I have always mentioned “it’s also about you and me, and it’s from the age of 40”. People often automatically think of 65* + *.” (R4, campaign coordinator).*

Furthermore, campaign coordinators and stakeholders mentioned that the majority of stakeholders who committed to becoming a “campaign friend” were affiliated with locations that are primarily visited by senior citizens, such as libraries, Alzheimer Cafés (accessible meeting locations where people affected by dementia and their caregivers can meet), and neighborhood associations.

#### Victim blaming and stigmatisation

The campaign slogan “We are the medicine ourselves” and the campaign posters were occasionally interpreted as stigmatising or victim-blaming, considering those who develop dementia later in life as self-responsible for their condition due to a poor lifestyle. To avoid potential stigmatisation and blaming the victim, it was necessary to provide a nuanced explanation of the campaign slogan.

*“The slogan “We are the medicine ourselves” can imply that you haven’t done enough to prevent dementia when you have dementia, and that it is therefore kind of your own fault. So I actually gave the instruction everywhere that you can reduce the chance that you will get it, but that does not change the fact that you can still get it.” (R15, campaign coordinator).*

Table 4 presents an overview of all implementation facilitators and barriers.

**Table 4:** Overview of implementation facilitators and barriers

Facilitators	Barriers
Existing campaign framework: availability of campaign website, MyBraincoach app, and campaign materials facilitated a quick start	Limited financial resources: budget constraints and unsuccessful funding applications hindered implementation
Evidence-based content and appealing campaign materials: the design and messaging were well-received by stakeholders	Impact of COVID-19 pandemic: restrictions limited in-person events and diverted public health funding
Early preparation of municipalities: clarifying expectations and roles ahead of time improved coordination	Sustainability challenges: uncertainty about how to continue the campaign beyond the funded period
Use of existing local networks and events: collaboration with ongoing initiatives helped amplify campaign reach	Difficulties engaging younger segment of the target group (40–60 years): activities mainly reached older adults
Involvement of local ambassadors: individuals with strong community ties helped reach underrepresented groups	Perceived victim blaming and stigmatization: the campaign slogan risked being interpreted as blaming individuals with dementia
Strong stakeholder networks: involvement of local partners, including campaign friends, supported visibility	Challenges recruiting active campaign friends: some partners expected mutual benefits that were not feasible

## Discussion

This study evaluated the public awareness campaign “We zijn zelf het medicijn”, conducted in nine regions in the Netherlands, and aimed to increase awareness of dementia risk reduction among middle-aged to older (40–75 years) community-dwelling individuals. The campaign reached 34% of the target population. Overall, pooled results showed that the campaign did not lead to a general increase in population-level awareness of dementia risk reduction. Yet, knowledge of specific modifiable dementia risk and protective factors and of two out of the three main campaign messages modestly increased over time. In addition, self-reported exposure to the campaign was associated with higher awareness, better recognition of modifiable risk and protective factors, and more self-reported lifestyle changes aimed at improving brain health. Interviews with campaign coordinators revealed several facilitators and barriers relevant to future campaign implementation.

Notably, our study found higher awareness among those who reported being exposed to the campaign, which is consistent with findings from previous awareness campaigns on dementia risk reduction conducted in Belgium and Denmark [7, 12] as well as our initial campaign in Limburg [9]. Campaign materials were more often recognised by individuals with lower and middle educational levels than by highly educated individuals, suggesting greater exposure among the former groups, though it did not translate to better awareness or knowledge. Underrepresentation of lower-educated individuals in the MHS survey panels may have limited the ability to detect changes in this group. Alternatively, the use of triggering questions on the campaign posters may not have effectively communicated the campaign message to individuals with lower educational levels. Interestingly, the initial Limburg campaign increased awareness among lower-educated individuals [9]. This finding aligns with our study, which showed that lower-educated participants were more likely to recognise campaign materials compared to higher-educated participants. This regional effect may be attributed to the lower baseline awareness in this group (18.2%) compared to the pooled baseline level (38.6%), providing greater potential for improvement.

The lack of significant population-level changes in awareness mirrors findings from the initial rollout in Limburg [9] and could be attributed to several factors. Many campaign coordinators faced limited budgets, unapproved funding requests, and, in some cases, redirection of financial resources to address the COVID-19 pandemic. Financial constraints limited the intensity of the campaign. Another challenge was engaging adults aged 40–60. Although the campaign deliberately messaged on brain health next to dementia risk, campaign activities were primarily attended by older individuals, and campaign friends were often affiliated with locations predominantly visited by senior citizens. Individuals aged 40–60 may not yet perceive themselves as at risk for dementia and prioritise other aspects of life, such as work and family, resulting in lower exposure to the campaign and engagement with the topic. This underscores the need for further research into communication preferences and engagement strategies of this group. The campaign slogan (“We are the medicine ourselves”) was framed to convey a positive and empowering message [9]. However, this slogan was occasionally perceived as victim-blaming, highlighting the importance of ethically responsible language when messaging about dementia risk reduction. Finally, the phrasing of the awareness statement used to assess awareness of dementia risk reduction ("There is nothing anyone can do to reduce their dementia risk") may have been overly complex. A simpler and more positively framed statement might have been more sensitive for measuring changes in awareness.

These challenges might have also led to the limited change in recognition of dementia risk and protective factors. In addition, it may be attributed to the campaign’s focused messaging, centering on three key messages: ‘eat healthy’, ‘exercise regularly’, and ‘stay curious’. Over time, recognition of two of these messages significantly increased, suggesting that a targeted approach can be effective in raising awareness. However, this narrow focus may have constrained broader improvements in recognition, as other modifiable dementia factors, many of which are well-known cardiovascular factors such as hypertension and hypercholesterolemia, received less emphasis. These conditions are often more complex and may be perceived as beyond individual control. By primarily emphasising lifestyle-related behaviours, there is a risk of unintentionally reinforcing a narrative of personal responsibility, potentially leading to stigmatisation or victim blaming, particularly among individuals living with chronic health conditions for which behaviour change may be more difficult or insufficient. A more comprehensive communication strategy may be needed to balance simplicity with inclusiveness and accuracy.

## Study strengths and limitations

Strengths of this study include the sizeable and independent samples of the quantitative survey. Additionally, the comprehensive surveys used have been administered across various populations to measure awareness [7–12, 23] and assess the effectiveness of public awareness campaigns aimed at improving awareness of dementia risk reduction [7, 9, 12]. This allows for direct cross-study comparisons and well-powered subgroup analyses. Notably, this study is among the first to evaluate both the effectiveness and the process of a public awareness campaign focused on dementia risk reduction.

This study also has limitations. The study samples were drawn predominantly from highly educated MHS survey panels, consisting of individuals proficient in Dutch and comfortable using the internet, probably resulting in selection bias. Additionally, the regional campaigns varied in characteristics, including the year of execution, duration, intensity, and contextual factors, including the COVID-19 pandemic. Although standardized pre- and post-surveys were employed, the surveys were administered independently by each region, with some adjustments in measurements and missing data. Campaign intensity was measured on a regional level based on the number of organised activities and public media posts per region. Detailed information on other channels (distribution of printed materials, website or app use, or earned media coverage) was not consistently available, preventing the construction of a composite intensity measure across modalities and the ability to explore dose–response effects. Future research should include systematic data collection across all dissemination channels and the development of validated, composite indices of campaign intensity.

## Recommendations for future campaigns

To enhance comprehension and reach among populations with limited health literacy, future campaigns should develop materials that clearly and accessibly communicate the positive impact of lifestyle choices on brain health. Messaging should emphasize supportive guidance while avoiding an overemphasis on individual responsibility. To maximise knowledge gains at the population level, future campaigns may benefit from prioritising lesser-known dementia risk and protective factors, such as cardiovascular and metabolic conditions. Campaigns addressing cardiovascular risk have demonstrated better uptake when risk communication is paired with actionable steps and clear referral pathways to local healthcare and lifestyle support sevices. Translating awareness into effective action requires providing practical follow-up options, such as primary care engagement and community lifestyle programmes [24].

Despite greater exposure to the campaign, participants with lower-to-middle education levels did not exhibit improved awareness or knowledge of dementia risk reduction compared to highly educated participants. Evidence from community-based interventions and small-media interventions in related public health prevention (e.g., obesity, cancer) indicates that culturally tailored, co-designed materials delivered through trusted community messengers effectively improve knowledge and service uptake [25]. Therefore, co-development of campaign materials with representatives from these communities is essential. Expanding inclusion to other underrepresented groups, such as individuals with migration backgrounds, will further enhance equity and impact.

To broaden campaign reach, collaborating with influential community figures and organisations is recommended. Persons with strong social networks and public presence can amplify messaging, particularly within under represented groups. A participatory bottom-up approach that actively involves communities in shaping and disseminating content fosters higher engagement and acceptance than top-down strategies [26].

Finally, integrating dementia risk reduction efforts into existing public health initiatives and leveraging their established networks and resources can enhance both campaign reach and sustainability.

## Conclusion

This multi-regional campaign did not lead to a general increase in population-level awareness of dementia risk reduction. Yet, knowledge of specific modifiable dementia risk and protective factors modestly improved over time. Furthermore, self-reported exposure to the campaign was associated with higher awareness of dementia risk reduction, better recognition of risk and protective factors, and more lifestyle-related behaviours aimed at improving brain health. Future campaigns are recommended to maximize the reach of younger individuals (aged 40–60), as well as individuals with lower educational levels and migration backgrounds. To maximise impact at the population level, co-creation, inclusive messaging, and collaboration with key community stakeholders are recommended.

## Supplementary Information

Supplementary Material 1.

## Data Availability

This will be made available on request from the corresponding author.

## Abbreviation

CI: Confidence Interval
COVID-19: Coronavirus disease 2019
LDL: Low-Density Lipoprotein
LIBRA: LIfestyle for BRAin health
MHS: Municipal Health Service
NDPI: Netherlands Dementia Prevention Initiative
SD: Standard Deviation
