# Supplementary material for: Evaluation of a public awareness campaign for dementia risk reduction in the Netherlands: a mixed methods study
Source: BMC Public Health. 2025 Dec 12;26:474. doi: 10.1186/s12889-025-25676-8 (PMC12874770; doi:10.1186/s12889-025-25676-8)
Supplement: Supplementary file 1 — Supplementary Material 1. [file 12889_2025_25676_MOESM1_ESM.docx]

**Supplementary Files**

**Article title**: Evaluation of a public awareness campaign for dementia risk reduction in the Netherlands: A mixed methods study

**Journal**: BMC Public Health

**Authors**: Dominique Paauw^1,2^, Irene Heger^1,2^, Dorothee Horstkötter^2,3^, Niels Janssen^1,2^, KlaasJan Hajema^4^, Sandra Kuiper^5^, Anne Loyen^6^, Judith Bouwmeester^7^, Anja Lens^8^, Judith Helmink^9^, Françoise Schütz^10^, Kay Deckers^1,2^, Sebastian Köhler^1,2^

**Affiliations**: ^1^Alzheimer Centre Limburg, Department of Psychiatry and Neuropsychology, Maastricht University, Maastricht, The Netherlands; ^2^ Mental Health and Neuroscience Research Institute (MHeNs), Maastricht University, Maastricht, The Netherlands; ^3^Department of Health Ethics and Society, Maastricht University, Maastricht, The Netherlands; ^4^GGD Zuid-Limburg, Heerlen, The Netherlands; ^5^GGD West-Brabant, Breda, The Netherlands; ^6^GGD Flevoland, Lelystad, The Netherlands; ^7^GGD Zuid-Holland Zuid, Dordrecht, The Netherlands; ^8^Plicare, Zoetermeer, The Netherlands; ^9^GGD Hart voor Brabant, Tilburg, The Netherlands; ^10^GGD regio Utrecht, Zeist, The Netherlands

**Correspondence to:** Sebastian Köhler, E: s.koehler@maastrichtuniversity.nl

**Supplementary File 1: Examples of campaign materials**

Campaign message ‘Exercise regularly’

English translation: “Does walking reduce the risk of dementia?”

Campaign message ‘Eat healthy’

English translation: “Do strawberries reduce the risk of dementia?”

Campaign message ‘Stay curious’

English translation: “Does learning Spanish reduce the risk of dementia?”

**Supplementary File 2: English translation of dementia awareness survey**

**Pre- and post-assessment**

-----------------------------------------------------------------------------------------------------------------

Demographics

1. How old are you?
   *[text field for number between 40 and 75]*
2. What is your gender?
   - Male
   - Female
   - Other
3. What is your zip code?
4. What is your marital status?
   - Married/registered partnership
   - Living together
   - Unmarried, never been married
   - Divorced
   - Widowed
5. What is your highest finalized degree of education?
   *[Six categories according to the Dutch education system, categorized into low, middle and high]*

-----------------------------------------------------------------------------------------------------------------

Dementia knowledge

1. Dementia describes a set of symptoms including loss of memory, sudden mood swings, not remembering who people are, and having trouble finding your words. Alzheimer's disease is one form of dementia. How much would you say you know about dementia?
   - A great deal
   - Quite a lot
   - Some
   - Not very much
   - Nothing at all
   - I don't know
   - I prefer not to answer this question

-----------------------------------------------------------------------------------------------------------------

Dementia risk awareness

Ple*ase state how much you agree or disagree with the following statements.*

1. ‘There is nothing anyone can do to reduce their risks of getting dementia’
   - Agree strongly
   - Agree
   - Neither agree nor disagree
   - Disagree
   - Disagree strongly
2. 'High blood pressure increases your chances of getting dementia'
   - Agree strongly
   - Agree
   - Neither agree nor disagree
   - Disagree
   - Disagree strongly
3. ‘Smoking increases your chances of getting dementia’
   - Agree strongly
   - Agree
   - Neither agree nor disagree
   - Disagree
   - Disagree strongly
4. ‘No or moderate alcohol use lowers your chances of getting dementia’
   - Agree strongly
   - Agree
   - Neither agree nor disagree
   - Disagree
   - Disagree strongly
5. ‘Regular physical activity lowers your chances of getting dementia’
   - Agree strongly
   - Agree
   - Neither agree nor disagree
   - Disagree
   - Disagree strongly
6. ‘Depression increases the chances of getting dementia’
   - Agree strongly
   - Agree
   - Neither agree nor disagree
   - Disagree
   - Disagree strongly
7. ‘Diabetes increases the chances of getting dementia’
   - Agree strongly
   - Agree
   - Neither agree nor disagree
   - Disagree
   - Disagree strongly
8. ‘Being overweight increases the chances of getting dementia’
   - Agree strongly
   - Agree
   - Neither agree nor disagree
   - Disagree
   - Disagree strongly
9. ‘A mentally active lifestyle lowers the chances of getting dementia’
   - Agree strongly
   - Agree
   - Neither agree nor disagree
   - Disagree
   - Disagree strongly
10. ‘Heart disease increases the chances of getting dementia’
    - Agree strongly
    - Agree
    - Neither agree nor disagree
    - Disagree
    - Disagree strongly
11. ‘Kidney disease increases the chances of getting dementia’
    - Agree strongly
    - Agree
    - Neither agree nor disagree
    - Disagree
    - Disagree strongly
12. ‘High cholesterol increases the chances of getting dementia’
    - Agree strongly
    - Agree
    - Neither agree nor disagree
    - Disagree
    - Disagree strongly
13. ‘Healthy diet lowers the chances of getting dementia’
    - Agree strongly
    - Agree
    - Neither agree nor disagree
    - Disagree
    - Disagree strongly
14. Would you be interested in receiving information on how to improve your brain health?
    - Yes
    - No
    - Maybe
15. In the case that there was a mobile application, providing you without charge with information about your brain health and giving advice on how to improve your brain health, would you use this app?

- Yes
- No
- Maybe

1. In the case that you would like to know more about your own brain health, what information channels would you use to find this information?
   *Select all that apply*
   - Search on the web
   - GP office
   - Webpage public health authorities
   - Website Dutch Alzheimer’s Association
   - Library
   - Other: [open *text field*]
   - None of these
   - I do not know
   - I prefer not to answer this question
2. In the case that you would decide to work on improving your own brain health, what could be an obstructing factor for you?
   *Select all that apply*
   - Lack of knowledge
   - Lack of time
   - Lack of company
   - Financial reasons
   - Lack of motivation
   - Difficult to organize
   - Financial reasons
   - Health problems
   - Other [*textfield*]
   - None of these
   - I do not know
   - I prefer not to answer this question

**Survey items included in the post-campaign survey only**

Exposure to the campaign

1. From *month/year* to *month/year*, the *X* ran a campaign on brain health and dementia prevention. Have you heard about this campaign?

- No
- Yes

1. Do you recognize the slogan “We are the medicine ourselves”?

- No
- Yes

1. Have you heard about the “MyBraincoach” app?

*[Illustrative screenshot of the app]*

- No
- Yes

1. Have you downloaded the “MyBraincoach” app?

- No
- Yes

1. Via what sources have you heard or seen something about prevention of dementia during the last year?

*Select all that apply*

- Television
- Radio
- Newspaper
- The campaign website
- Social media (Facebook, Instagram, Twitter, etc.)
- Campaign activitiy, such as a workshop or presentation
- I have not heard or seen something of this kind during the last year
- Other source: *[open text field]*

1. Have you seen these campaign materials during the last year?

*[Illustrative screenshots]*

- Campaign flyer [yes/no]
- Campaign poster [yes/no]
- Newsletter [yes/no]
- Socks [yes/no]
- Bookmark [yes/no]
- Card game [yes/no]

1. Have you adopted a healthier lifestyle during the past months/last year? If yes, what specific changes have you made?

*Select all that apply*

- I have not adopted a healthier lifestyle during the past months/last year
- Eat healthier
- Exercise more
- Consume less alcohol or stop drinking alcohol
- Smoke less or quit smoking
- Keep a curious mindset (playing a music instrument, learning a language, social activities)
- More relaxation or more adequate coping for depressive thoughts
- Monitor my glucose levels
- Monitor my weight
- Lowering my cholesterol
- Lowering my blood pressure
- Monitor my kidney function
- Monitor my heart condition

1. Did you adopt a healthier lifestyle during the past months/last year to improve your brain health?

- No
- Yes

1. Would you say that you have become more conscious of your brain health and the relationship of your brain health with your lifestyle over the past months/last year?

- No
- Yes

End

Thank you very much for filling in this questionnaire. If you have any remarks or questions regarding this study, please contact us via [*email address*].

**NOTE**

If you are interested to use this questionnaire, please contact: ([kay.deckers@maastrichtuniversity.nl](mailto:kay.deckers@maastrichtuniversity.nl)).

**Supplementary File 3: Semi-structured topic guides**

Campaign coordinators

**Theme 1: Role in the campaign**

1. Can you tell us more about what your role was during the “We are the medicine ourselves” campaign?

**Theme 2: Campaign message**

1. You have been involved in the “We are the medicine ourselves” campaign in MHS region X. If you were to describe the campaign in your own words, what would you say?

- What is the message of the campaign?

1. Do you think that this message was clear to the target audience (aged 40–75)?
2. Do you think that this message was clear to the parties involved in organizing the campaign (e.g. stakeholders like the municipality)?

- What were positive or negative experiences?

1. Were there campaign messages that resonated better than others? Eat healthy, exercise regularly, or stay curious?

**Theme 3: Campaign preparation**

1. What approach did you choose to prepare for the campaign (e.g., in terms of planning, budget, and selecting regions for implementation)?
2. How many people were involved in the preparation, and how much time did you approximately spend on it?

- Could you provide insight into your campaign budget?

1. Was there a particular focus on certain groups?
2. How were ethical dilemmas surrounding dementia prevention addressed during the campaign’s preparation, such as blaming-the-victim and stigmatisation?

**Theme 4: Positive aspects of the “We are the medicine ourselves” campaign execution**

1. What went well during the execution of the campaign in your region? Can you provide a few examples?

**Theme 5: Areas for improvement in the execution of the “We are the medicine ourselves” campaign**

1. What did not go well during the campaign execution?
2. What would you do differently when executing a new campaign?

**Theme 6: Engaging stakeholders in the “We are the medicine ourselves” campaign**

1. How did you reach out to parties to participate in the campaign?
2. Did you also try to recruit ambassadors for the campaign (these are well-known individuals with a large network)?

- If the answer is yes: What kind of individuals were these?

1. Were stakeholders enthusiastic about participating in the campaign activities?
2. Were there certain stakeholders who were not or less enthusiastic about participating in the campaign activities?

- If the answer is yes: Why did stakeholders not want to participate in the campaign activities?
- If the answer is yes: What attempts did you make to persuade these stakeholders to participate?

**Theme 7: Campaign materials for the “We are the medicine ourselves” campaign**

1. Which campaign materials were the most successful? Which materials reached the most people?
2. Did you develop campaign materials yourself?

- If the answer is yes: What were the costs, and what impact did these materials have?

1. Which campaign materials were the least successful?

- Why do you think these materials were less successful?

1. What do you think of the current design of the campaign materials (text and colours used)?

- Do you have any criticisms/suggestions for improvement?

1. Do you have suggestions for new campaign materials that could be used during a renewed execution of the “We are the medicine ourselves” campaign (booster campaign)?
2. Did you infirm people about the existence of the “MyBraincoach” app?

- If the answer is yes: What responses did you receive regarding the “MyBraincoach” app?

**Theme 8: Public responses to the ‘‘We are the medicine ourselves’ campaign**

1. What responses did you receive from the public?
2. Did members of the public provide any criticisms/suggestions for improvement of the campaign?

**Theme 9: Opportunity for additional input**

1. Is there anything else you would like to add to our conversation, or have we forgotten to ask you important questions?

Stakeholders

**Theme 1: Role in the campaign**

1. Can you tell us more about what your role was during the “We are the medicine ourselves” campaign?

**Theme 2: Campaign message**

1. You have been involved in the “We are the medicine ourselves” campaign in MHS region X. If you were to describe the campaign in your own words, what would you say?

- What is the message of the campaign?

1. Do you think that this message was clear to the target audience (aged 40–75)?
2. Were there campaign messages that resonated better than others? Eat healthy, exercise regularly, or stay curious?

**Theme 3: Positive aspects of the “We are the medicine ourselves” campaign execution**

1. What went well during the execution of the campaign in your region? Can you provide a few examples?

**Theme 4: Areas for improvement in the execution of the “We are the medicine ourselves” campaign**

1. What did not go well during the campaign execution?
2. Do you have any tips for other regions in the Netherlands that also want to roll out this campaign?

**Theme 5: Campaign materials for the “We are the medicine ourselves” campaign**

1. Which campaign materials were the most successful? Which materials reached the most people?
2. Which campaign materials were the least successful?

- Why do you think that these materials were the less successful?

1. What do you think of the current design of the campaign materials (text and colours used)?

- Do you have any criticisms/suggestions for improvement?

1. Do you have suggestions for new campaign materials that could be used during a renewed execution of the “We are the medicine ourselves” campaign (booster campaign)?
2. Did you infirm people about the existence of the “MyBraincoach” app?

- If the answer is yes: What responses did you receive regarding the “MyBraincoach” app?

**Theme 6: Public responses to the “We are the medicine ourselves” campaign**

1. What responses did you receive from the public?
2. Did members of the public provide any criticisms/suggestions for improvement of the campaign?

**Theme 7: Opportunity for additional input**

1. Is there anything else you would like to add to our conversation, or have we forgotten to ask you important questions?

**Supplementary File 4: Summary of the finalized themes and interpretations**

**Theme 1: Campaign preparation**

- Conduct surveys with residents to identify the themes they find important for healthy ageing
- Form a core group/project team
- Develop a campaign strategy: message, target audience, resources/materials, ambassadors/partners/friends, activities, and impact measurements
- Submit a grant application to the municipality
- Conduct a stakeholder analysis
- Gather campaign friends
- Set up a regional page on the campaign website

**Subtheme: Campaign budget**

- Budget varies by region
- Grant applications are usually rejected: challenging to gather funding
- Project leaders also use their own hours from general healthy ageing projects

**Subtheme: Project team**

- Composed of project leaders, project staff, alderman, civil servants, communication advisors, and the local dementia network
- The size of the project team varies greatly: sometimes 3 people, and sometimes 10 or more
- Enthusiasm and commitment within the project team are important

**Theme 2: Campaign message**

- Scientific background is useful to refer to
- Sufficient repetition is needed

**Subtheme: Campaign reach**

- The older target group (60–75 years) is mainly reached
- The campaign struggles to engage the younger target group (40–60 years): they do not feel addressed
- Mainly people already concerned with dementia (e.g., caregivers) show interest
- Focus on reaching vulnerable groups (low socio-economic position, migration background, limited health literacy)

**Subtheme: Campaign sustainability**

- It’s a pity to let go of the network of campaign friends after the campaign ends and not to reuse the materials
- Policy regarding the use of campaign materials after the campaign period is unclear: some regions consciously no longer use the materials, while others do by integrating them into other MHS themes (fall prevention, positive ageing, education)
- In some regions, there was a mini campaign after the campaign period, lasting a week/month or for example, on World Alzheimer’s Day

**Subtheme: Campaign slogan**

- Nuancing the campaign slogan is important: risk of stigmatization
- A powerful slogan
- Potential to extend it to other health topics

**Subtheme: Campaign messages**

- A good combination and useful as a guideline
- All three are seen as interesting
- The messages “Eat healthy” and “Exercise regularly” are well known
- The message “Stay curious” sparked the most interest

**Theme 3: Campaign materials**

- The materials were received very positively: simple and appealing
- Materials used:
  - Flyer
  - Banner
- Online banner
- Email banner
- Poster
- Video
- Newsletter
- Brain quiz
- Golf ball
- Billboard
- Vaccine box
- Pharmacist bag
- Waiting room screen
- Beer coaster

**Subtheme: Newly developed campaign materials**

- A5 flyer
- Turkish/Arabic flyers
- Desk calendars
- Pens
- T-shirt
- Socks
- Pennants
- Playing cards
- Bookmark
- Puzzle book
- Placemat

**Subtheme: Suggestions for new campaign materials**

- Tear-off pad for general practitioners
- Games
- Podcast
- Shopping cart tokens
- Foldable bags
- Mugs
- Lanyards

**Theme 4: Campaign activities**

- Kick-off event
- Webinar
- Lecture
- Workshop
- Walking tour
- Giant brain
- Closing event

**Subtheme: Social media**

- The campaign was mainly promoted on Facebook, LinkedIn and X
- Occasionally also on Instagram or via digital campaign banners on online pages
- A monthly content calendar was created for social media in coordination with a communications advisor and the campaign friends
- Sometimes paid advertisements were used, e.g., on Facebook

**Subtheme: Friends programme**

- The campaign message was enthusiastically received
- Mainly associations affiliated with older adults were interested in becoming campaign friends
- Snowball effect in recruiting campaign friends
- The level of involvement of campaign friends varied greatly
- Municipal policy staff were very cautious
- Use of campaign ambassadors, such as language ambassadors
- Associations/organisations sometimes expect something in return for participating as campaign friends
- Campaign newsletter for campaign friends

**Subtheme: Campaign website**

- The tool to edit the website was not user-friendly
- Many rules were attached to using the website
- The downloadable campaign materials on the website were very useful
- Maintaining the website was time-consuming
- The website incurred high costs

**Subtheme: MyBraincoach app**

- In practice, it was rarely promoted: not user-friendly due to the long initial questionnaire
- Less suitable for people with low to middle education levels and older adults with limited digital skills
- Positive feedback on the app’s design: the “cracking” walnut and the daily tips, facts, and recipes
- Few downloads of the app during the campaign period

**Supplementary File 5: Coding tree**

**Supplementary File 6: Overview of the campaign regions in the Netherlands**


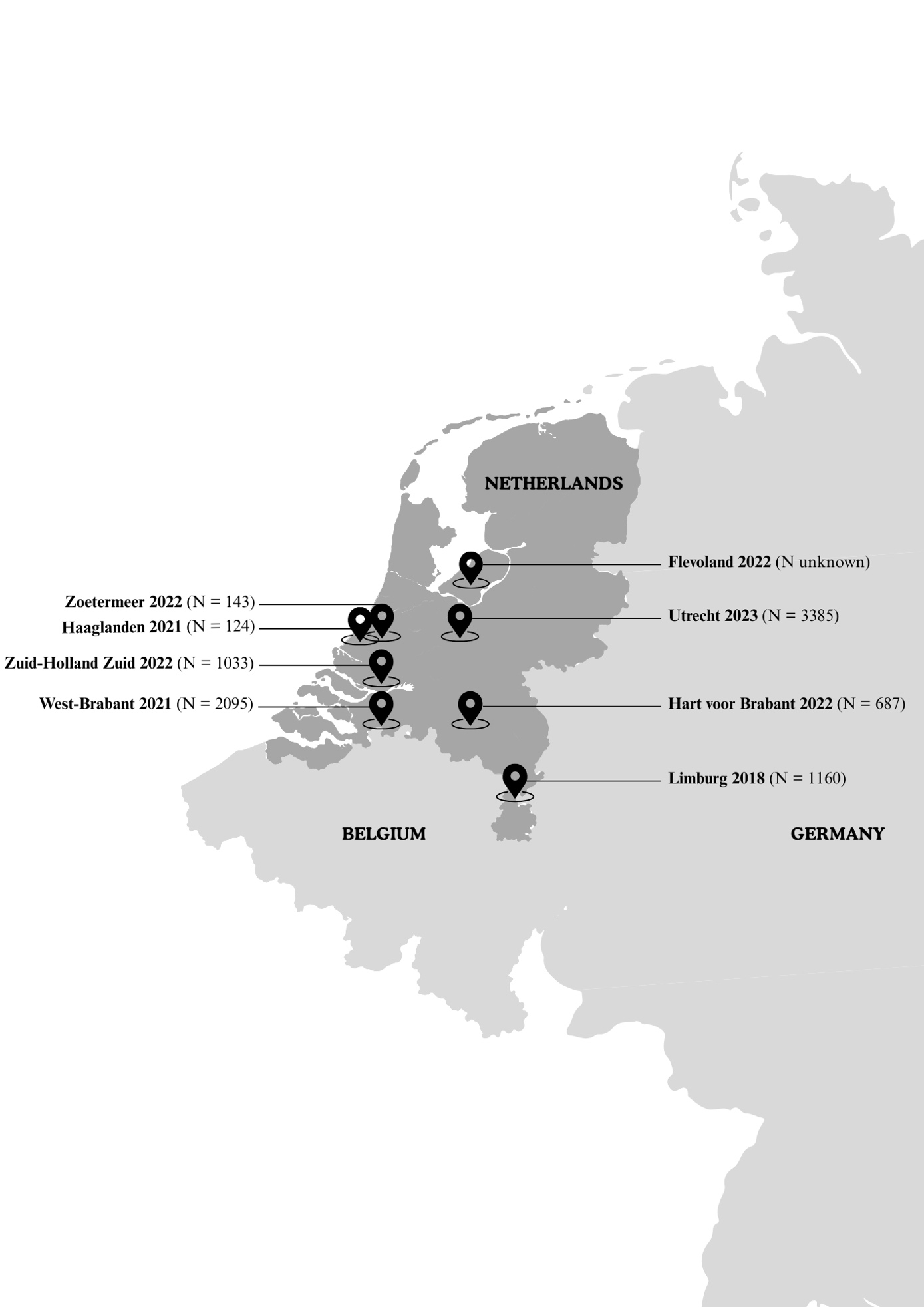


**Supplementary File 7: Pre-campaign results**

Before the campaign, 55.0% of respondents were aware of the potential of dementia risk reduction. People aged 40–60 years (B = -0.01, 95% CI: -0.01 – -0.00, p ≤ 0.001), higher educated people (X^2^ (2) = 190.03, p ≤ 0.001), and people with good self-reported knowledge of dementia (X^2^ (1) = 24.86, p ≤ 0.001) were more likely to be aware of dementia risk reduction compared to people older people, low- and middle-educated people, and people with poor self-reported knowledge of dementia.

The average number of correctly identified risk and protective factors was higher among people aged 40–60 years (B = -0.02, 95% CI: 0.03 – -0.01, p ≤ 0.001), highly educated people (low: 4.7, middle: 4.8, high: 5.9, p ≤ 0.001), and people with self-reported good knowledge of dementia (5.9 vs. 4.3; t (4854) = 10.61) (Fig. 3).

**Fig. 3** **Pre-comparison across educational groups**

Pre-campaign (n=4,981) comparison of the average number of correctly identified dementia risk and protective factors per Municipal Health Service region.*: significant difference compared to low-educated respondents (p < 0.05); **: significant difference compared to low-educated respondents (p < 0.01).

******

******

*****

The majority (58.4%) of respondents stated that they would like to receive more information on how to improve their brain health. People aged 40–60 years (B = -0.01, 95% CI: -0.01 – -0.00, p ≤ 0.001), men (63.6% vs. 53.9%; *X^2^ (4) = 51.56,* p ≤ 0.001), higher educated people (low: 53.7%, middle: 56.3%, high: 61.3%; *X^2^ (4) = 21.79,* p ≤ 0.001), and people with poor self-reported knowledge of dementia (63.0% vs. 57.5%; *X^2^ (2) = 15.55,* p ≤ 0.001) stated more often that they would like to receive more information on how to improve their brain health. Most respondents (50.4%) indicated they would *use a smartphone app to improve their brain health, with no statistically significant differences for age (B = 0.00, 95% CI: -0.00 – 0.01,* p = 0.584), gender (*X^2^ (2) = 4.77,* p = 0.092), educational level (*X^2^ (4) = 3.28,* p = 0.511), and self-reported knowledge of dementia (*X^2^ (1) = 3.92,* p = 0.141). Lack of knowledge was the most mentioned barrier to engage in a brain-healthy lifestyle (37.1%), followed by lack of motivation (16.5%) and lack of time (12.5%).

**Supplementary File 8: CROSS-checklist**

**Checklist for Reporting Of Survey Studies (CROSS)**

| **Section/topic** | **Item** | **Item description** | **Reported on page #** |
| --- | --- | --- | --- |
| **Title and abstract** | | |  |
| Title and abstract | 1a | State the word “survey” along with a commonly used term in title or abstract to introduce the study’s design. | 1 |
|  | 1b | Provide an informative summary in the abstract, covering background, objectives, methods, findings/results, interpretation/discussion, and conclusions. | 1 |
| **Introduction** | | |  |
| Background | 2 | Provide a background about the rationale of study, what has been previously done, and why this survey is needed. | 2 |
| Purpose/aim | 3 | Identify specific purposes, aims, goals, or objectives of the study. | 2 |
| **Methods** | | |  |
| Study design | 4 | Specify the study design in the methods section with a commonly used term (e.g., cross-sectional or longitudinal). | 4 |
|  | 5a | Describe the questionnaire (e.g., number of sections, number of questions, number and names of instruments used). | 5 |
| Data collection methods | 5b | Describe all questionnaire instruments that were used in the survey to measure particular concepts. Report target population, reported validity and reliability information, scoring/classification procedure, and reference links (if any). | 5 |
|  | 5c | Provide information on pretesting of the questionnaire, if performed (in the article or in an online supplement). Report the method of pretesting, number of times questionnaire was pre-tested, number and demographics of participants used for pretesting, and the level of similarity of demographics between pre-testing participants and sample population. | N/A |
|  | 5d | Questionnaire if possible, should be fully provided (in the article, or as appendices or as an online supplement). | 3-8 supplement |
| Sample characteristics | 6a | Describe the study population (i.e., background, locations, eligibility criteria for participant inclusion in survey, exclusion criteria). | 4 |
|  | 6b | Describe the sampling techniques used (e.g., single stage or multistage sampling, simple random sampling, stratified sampling, cluster sampling, convenience sampling). Specify the locations of sample participants whenever clustered sampling was applied. | 4 |
|  | 6c | Provide information on sample size, along with details of sample size calculation. | 4 |
|  | 6d | Describe how representative the sample is of the study population (or target population if possible), particularly for population-based surveys. | 4 |
| Survey  administration | 7a | Provide information on modes of questionnaire administration, including the type and number of contacts, the location where the survey was conducted (e.g., outpatient room or by use of online tools, such as SurveyMonkey). | 4 |
|  | 7b | Provide information of survey’s time frame, such as periods of recruitment, exposure, and follow-up days. | 4 |
|  | 7c | Provide information on the entry process:  –>For non-web-based surveys, provide approaches to minimize human error in data entry.  –>For web-based surveys, provide approaches to prevent “multiple participation” of participants. | 4 |
| Study preparation | 8 | Describe any preparation process before conducting the survey (e.g., interviewers’ training process, advertising the survey). | N/A |
| Ethical considerations | 9a | Provide information on ethical approval for the survey if obtained, including informed consent, institutional review board [IRB] approval, Helsinki declaration, and good clinical practice [GCP] declaration (as appropriate). | 7/8 |
|  | 9b | Provide information about survey anonymity and confidentiality and describe what mechanisms were used to protect unauthorized access. | 4 |
| Statistical  analysis | 10a | Describe statistical methods and analytical approach. Report the statistical software that was used for data analysis. | 6/7 |
|  | 10b | Report any modification of variables used in the analysis, along with reference (if available). | N/A |
|  | 10c | Report details about how missing data was handled. Include rate of missing items, missing data mechanism (i.e., missing completely at random [MCAR], missing at random [MAR] or missing not at random [MNAR]) and methods used to deal with missing data (e.g., multiple imputation). | 9 |
|  | 10d | State how non-response error was addressed. | 4 |
|  | 10e | For longitudinal surveys, state how loss to follow-up was addressed. | N/A |
|  | 10f | Indicate whether any methods such as weighting of items or propensity scores have been used to adjust for non-representativeness of the sample. | N/A |
|  | 10g | Describe any sensitivity analysis conducted. | N/A |
| **Results** | | |  |
| Respondent characteristics | 11a | Report numbers of individuals at each stage of the study. Consider using a flow diagram, if possible. | N/A |
|  | 11b | Provide reasons for non-participation at each stage, if possible. | N/A |
|  | 11c | Report response rate, present the definition of response rate or the formula used to calculate response rate. | 9 |
|  | 11d | Provide information to define how unique visitors are determined. Report number of unique visitors along with relevant proportions (e.g., view proportion, participation proportion, completion proportion). | 9 |
| Descriptive  results | 12 | Provide characteristics of study participants, as well as information on potential confounders and assessed outcomes. | 9/10 |
| Main findings | 13a | Give unadjusted estimates and, if applicable, confounder-adjusted estimates along with 95% confidence intervals and p-values. | 10 |
|  | 13b | For multivariable analysis, provide information on the model building process, model fit statistics, and model assumptions (as appropriate). | N/A |
|  | 13c | Provide details about any sensitivity analysis performed. If there are considerable amount of missing data, report sensitivity analyses comparing the results of complete cases with that of the imputed dataset (if possible). | N/A |
| **Discussion** | | |  |
| Limitations | 14 | Discuss the limitations of the study, considering sources of potential biases and imprecisions, such as non-representativeness of sample, study design, important uncontrolled confounders. | 20 |
| Interpretations | 15 | Give a cautious overall interpretation of results, based on potential biases and imprecisions and suggest areas for future research. | 20 |
| Generalizability | 16 | Discuss the external validity of the results. | 20 |
| **Other sections** | | |  |
| Role of funding source | 17 | State whether any funding organization has had any roles in the survey’s design, implementation, and analysis. | 23 |
| Conflict of interest | 18 | Declare any potential conflict of interest. | 22 |
| Acknowledgements | 19 | Provide names of organizations/persons that are acknowledged along with their contribution to the research. | 23 |

**Supplementary File 9: COREQ-checklist**

**COnsolidated criteria for Reporting Qualitative research**

| **Section/Topic** | **Item No** | **Checklist item** | **Reported on page No** |
| --- | --- | --- | --- |
| **Domain 1: Research team and reﬂexivity** | | | |
| Personal Characteristics | | | |
| *Interviewer/facilitator* | 1 | Which author/s conducted the interview or focus group?Interviewer/facilitator | 6 |
| *Credentials* | 2 | What were the researcher’s credentials? E.g. PhD, MD | 6 |
| *Occupation* | 3 | What was their occupation at the time of the study? | 6 |
| *Gender* | 4 | Was the researcher male or female? | 6 |
| *Experience and training* | 5 | What experience or training did the researcher have? Relationship with participants | 6 |
| Relationship with participants | | | |
| *Relationship established* | 6 | Was a relationship established prior to study commencement? | 6 |
| *Participant knowledge of the interviewer* | 7 | What did the participants know about the researcher? e.g. personal goals, reasons for doing the research | 6 |
| *Interviewer characteristics* | 8 | What characteristics were reported about the interviewer/facilitator? e.g. Bias, assumptions, reasons and interests in the research topic | 6 |
| **Domain 2: study design** | | | |
| Theoretical framework | | | |
| *Methodological orientation and*  *Theory* | 9 | What methodological orientation was stated to underpin the study? e.g. grounded theory, discourse analysis, ethnography, phenomenology, content analysis | 9 |
| Participant selection | | | |
| *Sampling* | 10 | How were participants selected? e.g. purposive, convenience, consecutive, snowball | 4/5 |
| *Method of approach* | 11 | How were participants approached? e.g. face-to-face, telephone, mail, email | 6 |
| *Sample size* | 12 | How many participants were in the study? | 4/5 |
| *Non-participation* | 13 | How many people refused to participate or dropped out? Reasons? | 4/5 |
| *Setting of data collection* | 14 | Where was the data collected? e.g. home, clinic, workplace | 6 |
| *Presence of non-participants* | 15 | Was anyone else present besides the participants and researchers? | 6 |
| *Description of sample* | 16 | What are the important characteristics of the sample? e.g. demographic data, date | 14 |
| Data collection | | | |
| *Interview guide* | 17 | Were questions, prompts, guides provided by the authors? Was it pilot tested? | Supplementary files |
| *Repeat interviews* | 18 | Were repeat interviews carried out? If yes, how many? | N/A |
| *Audio/visual recording* | 19 | Did the research use audio or visual recording to collect the data? | 5 |
| *Field notes* | 20 | Were ﬁeld notes made during and/or after the interview or focus group? | 5 |
| *Duration* | 21 | What was the duration of the interviews or focus group? | 5 |
| *Data saturation* | 22 | Was data saturation discussed? | 5 |
| *Transcripts returned* | 23 | Were transcripts returned to participants for comment and/or correction? | 7 |
| Domain 3: analysis and ﬁndings  Data analysis | | | |
| *Number of data coders* | 24 | How many data coders coded the data? | 7 |
| *Description of the coding tree* | 25 | Did authors provide a description of the coding tree? | Supplementary files |
| *Derivation of themes* | 26 | Were themes identiﬁed in advance or derived from the data? |  |
| *Software* | 27 | What software, if applicable, was used to manage the data? | 7 |
| *Participant checking* | 28 | Did participants provide feedback on the ﬁndings? | 8 |
| Reporting | | | |
| *Quotations presented* | 29 | Were participant quotations presented to illustrate the themes / ﬁndings? Was each quotation identiﬁed? e.g. participant number | 15-18 |
| *Data and ﬁndings consistent* | 30 | Was there consistency between the data presented and the ﬁndings? | 15-18 |
| *Clarity of major themes* | 31 | Were major themes clearly presented in the ﬁndings? | 15-18 |
| *Clarity of minor themes* | 32 | Is there a description of diverse cases or discussion of minor themes? | 15-18 |
